# Ecological Uptake and Depuration of Carbon Nanotubes by *Lumbriculus variegatus*

**DOI:** 10.1289/ehp.10883

**Published:** 2008-01-16

**Authors:** Elijah J. Petersen, Qingguo Huang, Walter J. Weber

**Affiliations:** 1 Department of Chemical Engineering, University of Michigan, Ann Arbor, Michigan, USA; 2 Department of Crop and Soil Sciences, University of Georgia, Griffin, Georgia, USA

**Keywords:** bioaccumulation, carbon nanotubes, ecotoxicology, environmental risks, fullerenes, *Lumbriculus variegatus*, MWNT, nanomaterials, nanotechnology, SWNT

## Abstract

**Background:**

Carbon nanotubes represent a class of nanomaterials having broad application potentials and documented cellular uptake and ecotoxicological effects that raise the possibility that they may bioaccumulate in living organisms.

**Objectives:**

Radioactively labeled nanotubes were synthesized using a novel methane chemical vapor deposition procedure. Single-walled carbon nanotubes (SWNTs), multiwalled carbon nanotubes (MWNTs), and pyrene were spiked to sediment samples, and the respective uptake and depuration of these nanotubes and pyrene were assessed by the oligochaete, *Lumbriculus variegatus*.

**Results:**

^14^C-labeled carbon nanotubes were developed for these experiments to overcome significant previous limitations for quantifying nanotube materials in environmental and biological media. Biota-sediment accumulation factors for SWNTs and MWNTs were observed to be almost an order of magnitude lower than those for pyrene, a four-ringed polycyclic aromatic hydrocarbon (PAH). The depuration behaviors of the oligochaete suggested that the nanotubes detected in these organisms were associated with sediments remaining in the organism guts and not absorbed into cellular tissues as was the pyrene. The results suggest that, unlike PAHs, purified carbon nanotubes do not readily absorb into organism tissues.

Carbon nanotubes have been the subject of extensive research over the past decade because of potential breakthroughs in a broad range of applications. Discovered by Sumio Iijima in 1991 (Iijima 1991), nanotubes are essentially seamless cylinders composed of sp^2^-hybridized carbon atoms arranged in a regular hexagonal pattern. Single-walled nanotubes (SWNTs) and multiwalled nanotubes (MWNTs) make up the two principal classes of carbon nanotubes. SWNTs are one-layer graphitic cylinders having diameters on the order of a few nanometers, whereas MWNTs are composed of numerous concentric cylinders having much larger diameters.

Although carbon nanotubes have drawn widespread research attention in recent years, their potential environmental and human health impacts have not been well characterized, and the risks they may pose to the welfare of humankind and the environment are largely unknown (Colvin 2003). A number of studies have indicated that carbon nanotubes can, in fact, enter cells (Cherukuri et al. 2004; Heller et al. 2005; Kam et al. 2004, 2006; Kostarelos et al. 2007; Monteiro-Riviere et al. 2005) and cause toxic damage to cells (Pulskamp et al. 2007; Sayes et al. 2006; Shvedova et al. 2003) and to aquatic organisms (Cheng et al. 2007; Roberts et al. 2007; Smith et al. 2007; Templeton et al. 2006). SWNTs have been detected qualitatively in *Daphnia magna* (Roberts et al. 2007), the estuarine copepod *Amphiascus tenuiremis* (Templeton et al. 2006), and the fish *Oncorhynchus mykiss* (Smith et al. 2007), although the extent to which nanotubes accumulate in these organisms was not quantitatively ascertained. One potential approach for predicting such behaviors is via comparison with hydrophobic organic chemicals that share some chemical similarities, such as polycyclic aromatic hydrocarbons (PAHs), counterparts of smaller sizes having between two to seven aromatic rings. PAHs accumulate readily in the fatty tissue of organisms, in large part as a result of the combined hydrophobicities and resistances of these chemicals to microbial degradation (Di Toro et al. 1991; Jager et al. 2003). Taken in combination with the observed facile cellular uptake of nanotubes and their detection in aquatic organisms, this leads to the conjecture that carbon nanotubes may also be bio-accumulable entities. This would, of course, have profound implications for ecological and human health. If organisms do uptake carbon nanoparticles, these materials, like PAHs, might also be transferred through food chains and enter organisms, such as humans, at higher trophic levels in significant amounts. In addition to the risks posed by the carbon nanotubes themselves, these materials strongly adsorb organic and inorganic chemicals and may exacerbate the biological uptake of such pollutants in environmental systems (Li et al. 2003; Yang et al. 2006).

A substantial challenge that has limited investigation of nanotube behaviors in environmental settings is the lack of a method by which to quantify them in biological or environmental media. The polydispersivity of nanotube mixtures with a broad range of diameters and lengths hinders the use of chromatographic techniques. Methods based on elemental analyses and spectroscopic techniques are generally not feasible because of the presence of organic matter. Spectrofluorimetry is one approach that has been used successfully to quantify nanotubes in cells and rabbits (Cherukuri et al. 2004, 2006). Given the unknown typical aggregation state of nanotubes in environmental systems, this technique has limited potential because of its inability to detect metallic SWNTs or nanotube bundles with metallic SWNTs (O’Connell et al. 2002). Raman spectroscopy was used to detect SWNTs qualitatively in the aquatic organism *D. magna* (Roberts et al. 2007). This approach, however, cannot provide quantitative results and is best suited for only SWNTs. A method used recently to detect nanotubes in biological systems is tagging them with molecules that are either bonded to radioactive isotopes or are themselves fluorescent (Kam et al. 2004, 2006; Singh et al. 2006). However, the use of such probes depends on the stability of its attachment to the nanotubes and, for radioactive labeling, attachment of the isotope to the polymer. The addition of such bulky tags likely influences the physicochemical properties of the nanotubes and thus their environmental behaviors.

To allow accurate evaluation of the extent to which carbon nanotubes released to the environment may bioaccumulate in organisms, we synthesized ^14^C-labeled carbon nanotubes (both single- and multiwalled) using modified chemical vapor deposition procedures. These nanotubes were spiked to sediments and assessed with respect to their uptake by *Lumbriculus variegatus*, a sediment-burrowing oligochaete. Oligochaetes have been used extensively as bioindicators of pollution (Lauritsen et al. 1985), and *L. variegatus* has been identified by the U.S. Environmental Protection Agency (EPA) as the freshwater organism of choice for assessing bioaccumulation (U.S. EPA 2000).

## Materials and Methods

### Chemicals

Nickel nitrate hexahydrate (99%), magnesium nitrate hexahydrate (reagent grade), ferric nitrate (reagent grade), and citric acid were purchased from Fisher Scientific (Fair Lawn, NJ). Alkaline magnesium carbonate was obtained from Sigma Aldrich (Sleeze, Germany). ^14^C-methane was obtained from American Radiolabeled Chemicals (St. Louis, MO). Helium gas (99.95%), argon gas (99.998%), and methane gas (99.97%) were from Cryogenic Gases (Detroit, MI).

### Carbon nanotube synthesis and purification

MWNTs were synthesized by a modified chemical vapor deposition with mixtures of ^14^C-labeled methane and regular methane as feedstock gases (Chen et al. 1997). We mixed 1.94 g nickel nitrate, 2.56 g magnesium nitrate, and 2 g citric acid, then added 20 mL Milli-Q water (Millipore Corp., Billerica, MA). This solution was dried at 100°C, and the solid was calcined at 700°C for 5 hr. One hundred milligrams of this catalyst was added to a quartz boat, and hydrogen gas was passed over the boat as the temperature in the reactor was raised to and held at 600°C. The flow of hydrogen gas was then stopped, and a mixture of radioactive and regular methane was introduced at a flow rate of 300 mL/min for 30 min. After the methane gas flow was stopped, the reactor was cooled to room temperature in Ar.

SWNTs were similarly synthesized using a methane chemical vapor deposition method with a catalyst composed of iron on a magnesium oxide support matrix (Li et al. 2002). Alkaline magnesium carbonate was annealed under Ar at 400°C for 1 hr. One gram of iron nitrate was dissolved in 100 mL MilliQ water and mixed with 10 g annealed magnesium carbonate. This solution was bath sonicated for 30 min and dried; the solids were ground to a powder with mortar and pestle. One gram of the catalyst was heated to 850°C under a stream of 250 mL/min Ar, then a mixture of regular and ^14^C methane flowing at 60 mL/min mixed with 250 mL/min Ar gas was passed over the catalyst for 15 min before cooling in Ar. The SWNTs and MWNTs were each purified by bath sonication for 1 hr in 10 M hydrochloric acid (Fisher Scientific).

### Carbon nanotube characterization

Carbon nanotubes were analyzed microscopically by transmission electron microscopy (TEM). Samples were prepared by adding dispersed nanotubes onto holey carbon film grids (Ted Pella, Redding, CA) and viewing the grids using a 3011 TEM (JEOL Ltd., Peabody, MA) operating at 300 kV. Thermal gravimetric analysis (TGA) (Pyris 1 TGA; Perkin Elmer, Waltham, MA) was also used to analyze the nanotube samples with respect to the presence of amorphous carbon impurities and residual catalyst materials. Because of the less stable chemical structure of amorphous carbon impurities, their presence can be assayed by analyzing derivatives of mass change with respect to temperature; in addition to the principal peak representative of the carbon nanotubes, a peak at a lower temperature represents the oxidation of carbon impurities. The percentage of original mass remaining after oxidation represents the fraction of residual catalyst in the sample. Raman spectra were obtained using a Renishaw inVia Raman microscope (Renishaw Inc., Chicago, IL) equipped with a Leica microscope, RenCam CCD detector, 785 nm diode laser, 1,200 lines/mm grating, and 50-μm slit. The radioactivity of the nanotube samples was assessed using biological oxidation (OX 500; R.J. Harvey Instrument Corp., Tappan, NY). Determination of the nanotube radioactivity by their direct addition to scintillation cocktail underestimated the nanotube radioactivity compared with combustion in a biological oxidizer, perhaps as a result of the absorption of beta emissions in nanotube bundles.

### Oligochaete culturing

*L. variegatus* obtained from Carolina Biological Supply Co. (Burlington, NC) were used to assess carbon nanotube availability to biological uptake and accumulation. The organisms were cultured in aquaria containing artificial freshwater [International Standards Organization (ISO) 1996] and unbleached brown paper towels and was maintained at 21 ± 2^o^C under photo-period (light:dark) ratios of 16:8 hr. We changed the overlying water and fed aquatic worms (daphnia food; Carolina Biological Supply Co.) at least 2 times per week.

### Sediments

Carbon nanotubes and pyrene were added separately to either mixtures of 90% sediment (Huron River, Ann Arbor, MI) with 10% Michigan (MI) Peat (by mass) or to un-amended sediment. The addition of 10% MI Peat allowed for a larger number of worms to be used for the bioavailability experiments, with a 50:1 ratio of sediment organic carbon to dry weight of the aquatic worms (Kukkonen and Landrum 1994). Sediment and peat samples were analyzed to ensure that neither contained any traces of the target contaminants. The sediment was air dried and passed through a 2-mm mesh sieve prior to ecological experiments. The organic carbon contents of the sediment and peat were 0.66% and 45.1%, respectively.

### Uptake experiments

Uptake experiments were conducted according to a modified U.S. EPA method (U.S. EPA 2000). ^14^C-SWNTs (0.03 or 0.003 mg/g dry sediment) and MWNTs (0.37 or 0.037 mg/g dry sediment) were dispersed by sonication in water prior to addition to the sediment. ^14^C-labeled pyrene (positions 4,5, 9, and 10) in methanol and nonradioactive pyrene were dissolved in acetone and added to sediment to give a final mass ratio of 0.054 mg/g dry sediment. The samples were thoroughly tumbled, and the acetone from the pyrene samples allowed to volatilize, then the samples were refrigerated. Sediment samples were freeze dried, combusted using the biological oxidizer, and radioactivity was measured by scintillation counting to determine initial concentrations of materials in the sediments and the homogeneities of their distributions. Elevated nanotube concentrations were detected occasionally, likely as a result of carbon nanotube aggregates not being fully dispersed during sonication. Samples for which sediment radioactivities were greater than 2 times mean values were excluded from calculations of mean sediment concentration. Sediment samples spiked with nonradioactive carbon nanotubes or pyrene and unspiked sediment samples were prepared as controls.

Six days after spiking with carbon nanotubes or pyrene, a 50-g (dry weight) quantity of amended or unamended sediment sample was added to 300-mL lipless beakers, and twice-daily artificial freshwater renewal was initiated (ISO 1996). Aquatic worms were removed and placed in a tray for 1 day prior to the start of each experiment. On the following day, 60 aquatic worms were added to each container to achieve a ratio of approximately 50:1 organic carbon in the sediment to dry mass of aquatic worms (Kukkonen and Landrum 1994). The worms were not fed during the experiment. At the beginning of an experiment and on a weekly basis thereafter, measurements of hardness, pH, dissolved oxygen, alkalinity, and conductivity were made to ensure that the water quality remained relatively constant during the experiments (U.S. EPA 2000).

We sieved aquatic worms from the sediments after predetermined intervals to determine the uptake of desired entities. The worms were collected from the sediment and placed in beakers with 500 mL of new artificial freshwater for 6 hr, a period shown to allow these organisms to purge > 98% of their gut content but also minimize tissue depuration of non-polar hydrophobic chemicals (Mount et al. 1999). The worms were blotted dry, weighed, and added to biological oxidizer boats with 100 mg d-mannitol to aid combustion. After drying overnight, the worms were combusted in the biological oxidizer and radioactivity was measured by liquid scintillation counting.

On days 7, 14, and 28, aquatic worms were also removed from containers with non-radioactive nanotubes or pyrene and unmodified sediments. We compared the number of living worms in these containers and those with ^14^C-labeled compounds. Lipid content was measured using a spectrophotometric method for the aquatic worms from nanotube-and pyrene-spiked sediments (Van Handel 1985). Biota–sediment accumulation factors (BSAFs) were calculated as the ratio of the concentration of a substance in an organism normalized by the organism lipid fraction to its concentration in the sediment normalized by its organic carbon fraction.

### Depuration experiments

On day 14 or 28, aquatic worms from three containers were added either to 600-mL beakers containing 500 mL clean water or to 300-mL lipless beakers containing 50 g dry mass clean sediment and filled with clean water. Worms added to clean sediment were removed from containers after a predetermined depuration interval and sediment particles were removed. After depuration for 1, 2, or 3 days, the worms were removed from their containers, blotted dry, then added to biological oxidizer boats with 100 mg d-mannitol. Radioactivities remaining in the worms were determined via biological oxidation and scintillation counting as described above.

## Results

**Figure 1:**
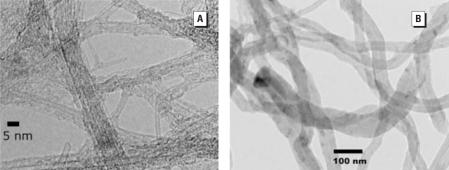
TEM of carbon nanotubes. Micrographs of (*A*) single-walled carbon nanotubes and (*B*) multi-walled carbon nanotubes.

**Figure 2:**
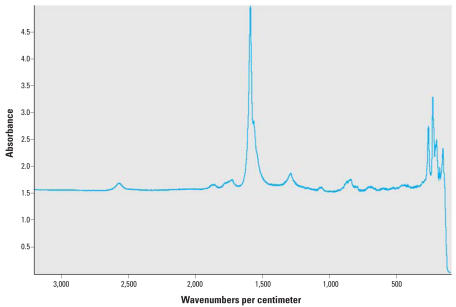
Raman spectrograph for single-walled carbon nanotubes. This spectrum is the average of nine measurements taken at different locations on the carbon nanotube sample.

**Figure 3:**
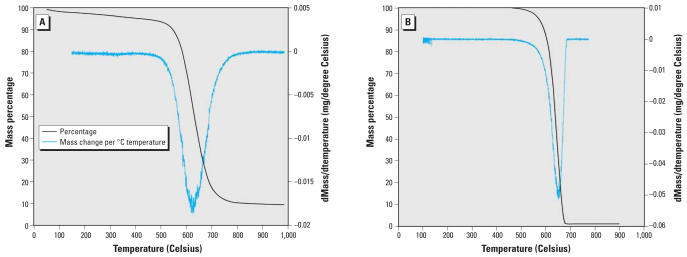
Thermal gravimetric analysis graphs for purified (*A*) single-walled carbon nanotubes and (*B*) multiwalled carbon nanotubes.

TEM micrographs indicate high purity nanotube samples with amorphous carbon rarely noted (Figure 1). Nanotube lengths ranged from hundreds of nanometers to a few micrometers. Diameters of the MWNTs ranged from 30 to 70 nm, whereas those for the SWNTs typically ranged from 1 to 2 nm, a reasonable result given the respective structural characteristics of these types of nanotubes. As shown in Figure 2, Raman spectroscopy revealed a high ratio of the G band to the D band, thus indicating the high purity of the SWNT sample with regard to amorphous carbon (Itkis et al. 2005), a particularly important characteristic given the radioactive-labeling approach used. The purities of the nanotubes with respect to amorphous carbon and catalyst impurities were assayed using thermal gravimetric analysis (Figure 3). The low mass remaining after combustion indicates the high purity of the nanotube samples. More specifically, the carbon percentages of the purified SWNTs and MWNTs were 92% ± 0.4 and 99% ± 1, respectively. Additional treatment processes could have removed higher fractions of catalyst impurities for the SWNT samples, but a prohibitively large mass of nanotubes would be lost in the additional processing steps, given the high cost of the radioactive methane. Additionally, the purity of the SWNT sample purities was judged sufficiently high for evaluation of bioaccumulation potential. Concentrations of amorphous carbon impurities for the purified MWNT and SWNT samples were minimal, as indicated by the absence of second peaks in the plots of rate of change of the mass versus heating time. Specific radioactivities of the purified SWNTs and MWNTs were 1.35 ± 0.03 mCi/g and 0.122 ± 0.004 mCi/g, respectively. The low standard deviations of specific radioactivity values suggest a relatively uniform distribution of ^14^C atoms.

Increases in the mortality of *L. variegatus* exposed to sediments containing SWNTs, MWNTs, or pyrene relative to unspiked sediments were not observed at the concentrations and exposure durations investigated. Measurement of acute toxicity across a broad range of nanotube concentrations was not attempted.

**Figure 4:**
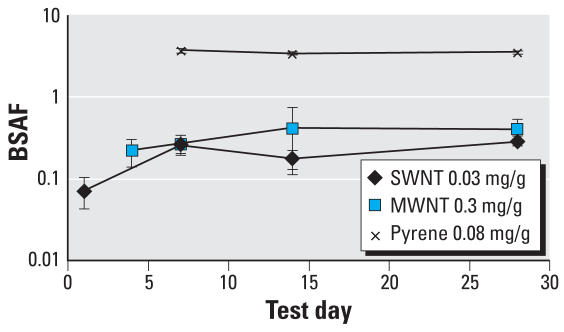
Carbon nanotube and pyrene uptake by *L. variegatus*. BSAFs of SWNTs (0.03 mg/g dry sediment), MWNTs (0.37 mg/g dry sediment), and pyrene (0.054 mg/g dry sediment) uptake by *L. variegatus*. All compounds were spiked to mixtures of 90% sediment (Ann Arbor, MI) with 10% MI Peat (by mass). Error bars represent SD ± 1 (*n* = 3).

Despite compositions of fused benzene rings similar to those of pyrene, BSAFs for *L. variegatus* for the SWNTs and MWNTs were almost an order of magnitude lower than those for pyrene, as illustrated in Figure 4. The uptake data do not indicate systematic differences between the SWNTs and MWNTs. The SWNTs may have been present as bundles, as indicated in Figure 1, with apparent diameters approaching those of the MWNTs, thus effecting similar uptakes. BSAF values for worms exposed to sediments spiked with SWNTs, MWNTs, and pyrene for 28 days were 0.28 ± 0.03, 0.40 ± 0.1, and 3.6 ± 0.2, respectively. BSAF values for 16 different PAHs having broadly varying hydrophobicities exposed to sediments for 28 days and with a depuration interval of 12 hr range from 0.4 to 5 (Ingersoll et al. 2003), thus confirming low uptake values for carbon nanotubes relative to those for PAH compounds. We performed subsequent experiments in which the organic carbon content of the sediment was decreased by a factor of eight by not adding the MI Peat amendments. In these experiments a decrease in the BSAF value from 0.51 ± 0.09 to 0.035 ± 0.015 was observed after 14 days of exposure. Additionally, decreasing the MWNT and SWNT concentrations spiked to the sediments by an order of magnitude did not significantly change the BSAF values.

**Figure 5:**
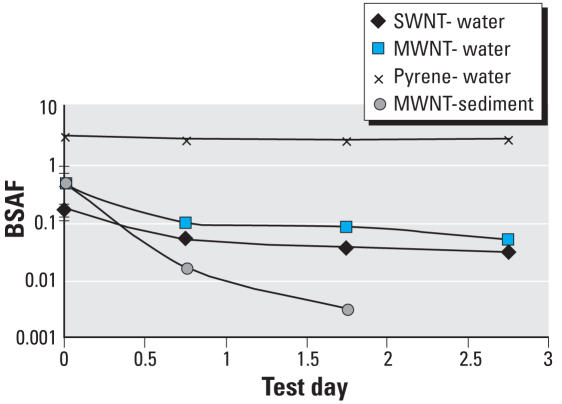
*L. variegatus* depuration of SWNTs, MWNTs, and pyrene after 14-day exposure. BSAFs of SWNTs (0.03 mg/g dry sediment), MWNTs (0.37 mg/g dry sediment), and pyrene (0.054 mg/g dry sediment) by *L. variegatus*. “Water” indicates samples for which the depuration was conducted in beakers with only water; “sediment” indicates that the depuration was conducted in beakers with water and 50 g clean sediment. Times represent the depuration period after the standard 6 hr for gut clearance. Error bars for day 0 represent SD ± 1 (*n* = 3).

We also investigated how rapidly the organisms purged carbon nanotubes (Figure 5). After approximately 3 days of depuration in beakers containing only water, the organisms had purged over 80% of single- or multiwalled nanotubes remaining after the initial 6 hr of depuration, whereas only 13% of the pyrene was excreted after the same interval. Depuration rates of MWNTs in beakers containing both water and clean sediment were significantly greater than those in beakers containing only water, suggesting that the worms would almost completely purge carbon nanotubes after a few days of exposure to clean sediments. Concentrations of nanotubes detected in organisms were below background concentration levels after 2 days of depuration in clean sediment dispersions in water. This more rapid depuration of carbon nanotubes for organisms in systems with both water and clean sediment is in accord with behaviors found for PAHs in previous studies (Landrum et al. 1994).

## Discussion

Assuming that partitioning processes leading to an eventual bioaccumulation of nanotubes by organisms depend on establishment of a thermodynamic equilibrium condition between sediment organic carbon and organism lipid phases, the fraction of organic carbon in the sediment would not be expected to affect BSAF values for nonionic organic chemicals (Di Toro et al. 1991). This is in contrast to the significant differences in BSAF values between nanotubes in unamended sediments and those in sediments containing 10% MI Peat observed here. These findings suggest that the nanotubes detected have not been absorbed into organism tissues but rather are associated with sediment matter remaining in the gut of the organism.

Interestingly, standard deviations of BSAF values for carbon nanotubes are significantly larger than those for pyrene. This result may support the notion that a significant fraction of the radioactivity detected in the aquatic worms was from sediment-associated nanotubes not purged from the organisms after 6 hr of depuration, a parameter that would reasonably vary over a greater range than that of absorption by tissues. This variability may also stem from greater heterogeneities of carbon nanotube distributions in the sediment. Although all pyrene was dissolved in acetone prior to spiking, some carbon nanotubes may not have been fully dispersed by sonication. Larger aggregates of carbon nanotubes may then have caused small regions of elevated nanotube mass concentration.

The depuration behaviors of nanotubes and pyrene also suggest different distribution patterns in the organisms. The relatively slow depuration of pyrene is attributed to slow rates of clearance from organism tissues compared with rates of sediment gut purging. Conversely, rapid elimination of carbon nanotubes suggests that the major fraction of carbon nanotubes present in the worms after an initial 6 hr of depuration consisted of nanotubes associated with residual gut sediment, an explanation in accord with results from the bioaccumulation experiments. One possible approach in estimating the concentration of nanotubes in the guts of oligochaetes is the work of Mount and co-workers (1999), who determined that the concentration of sediment in the guts of the organisms decreased from approximately 0.14% after 6 hr of depuration to 4.3 × 10–7% after 24 hr. As such, the BSAF values in the worms after 24 hr of depuration may be taken as an estimate of the concentration in the worms not associated with the gut contents.

Differences between the uptake and depuration behaviors of carbon nanotubes and pyrene might be attributable to several factors. *L. variegatus* can uptake sediment via three routes: pore water, overlying water, and ingestion of sediments. One possible explanation is that, unlike pyrene, carbon nanotubes are not present at significant concentrations in either pore or overlying waters. Radioactivity was not detected in overlying waters during any of the exposure experiments, suggesting that uptake of carbon nanotubes through overlying and pore water routes was minimal. This behavior could be a result of nanotube insolubilities or their strong sorption to sediment organic matter. Negligible increases in BSAF values for the nanotubes after the first day, however, suggests that an apparent equilibrium (or steady state) was rapidly reached because of lack of absorption in the lipids of the organisms.

The sizes of carbon nanotubes may be another factor in the lack of absorption by organisms. Size-dependent toxicities of SWNTs have been shown previously for the copepod *A. tenuiremis* (Templeton et al. 2006), and the uptake of the polybrominated diphenyl ether congener BDE 209 is significantly lower than that for other smaller and less hydrophobic congeners (Ciparis and Hale 2005). The low uptake of this congener was speculated to be a result either of its minimal desorption from sediment particles into pore waters or, after ingestion, into organism intestinal fluids, or from the large molecular size of the compound hindering cellular uptake. Cellular uptake of single- and multiwalled carbon nanotubes has been found in numerous studies (Cherukuri et al. 2004; Heller et al. 2005; Kam et al. 2004, 2006; Kostarelos et al. 2007; Monteiro-Riviere et al. 2005), but the extent to which nanotubes can travel across organisms tissues is unknown. Nanotubes may simply accumulate on the outermost cells of organisms and then be removed periodically when those cells are sloughed, or they may be able to pass through these cells and eventually enter systemic circulation in organisms. Furthermore, different nanotube synthesis techniques and physical or chemical modifications to the nanotubes may profoundly influence their biodistribution in organisms. Additional research to understand such nanotube interactions in biological systems and to link toxicologic impacts to nanotube concentrations in organisms is essential, and the ^14^C nanotubes developed here are ideally suited for such investigations.

Beyond the risks posed by nanotubes themselves, it is entirely possible that such materials may influence the bioaccumulation and fate of other pollutants in environmental systems. Carbon nanotubes possess strong sorptive capacities for such metals as lead, cadmium, and copper and various hydrophobic organic chemicals (Li et al. 2003; Yang et al. 2006). Hypothetically, carbon nanotubes may act in a manner similar to charcoal and other forms of black carbon by sequestering such compounds and limiting their bioavailability and mobility. It is also possible that nanotubes could serve as concentrators, durable sources, and transporters of such chemicals into organisms, thus exacerbating bioaccumulation and food chain transfer. Although nanotubes were not shown here to accumulate within oligochaetes, the passage of materials loaded with elevated concentrations of toxic chemicals through organisms could pose serious environmental and human health risks. Elucidating these effects represents another critical future research direction.
